# Fungal Transformation of Tree Stumps into a Suitable Resource for Xylophagous Beetles via Changes in Elemental Ratios

**DOI:** 10.3390/insects7020013

**Published:** 2016-04-09

**Authors:** Michał Filipiak, Łukasz Sobczyk, January Weiner

**Affiliations:** Institute of Environmental Sciences, Jagiellonian University, ul. Gronostajowa 7, 30-387 Kraków, Poland; lukasz.sobczyk@uj.edu.pl (L.S.); january.weiner@uj.edu.pl (J.W.)

**Keywords:** ecological stoichiometry, fungi, decomposition, trophic link, nutrient flow, deadwood, beetle, xylophage, nutrition, development

## Abstract

The elements present in dead pine stumps inhabited by larvae of wood-boring beetles (*Stictoleptura rubra*, *Arhopalus rusticus* and *Chalcophora mariana*) were analyzed over the initial (first 5 years; a chronosequence) stages of wood decay. The quantities of N, P, K, Ca, Mg, Fe, Zn, Mn, Cu and Na (but not S) increased with increases in the content of ergosterol (used as a proxy for the amount of fungal tissue). In fact, the amounts of P, N, K, Fe and Cu presented marked increases. These findings show that fungi stoichiometrically rearrange dead wood by importing externally occurring nutrients to decaying stumps. During the first years of wood decay, the ratios of C to other elements decrease substantially, but differently, for various elements, whereas the *N:Fe*, *N:Cu*, *N:P* and *N:K* ratios remain relatively stable. Therefore, the stoichiometric mismatch between xylophages and their food is greatly reduced. By changing the nutritional stoichiometry of dead wood, fungi create a nutritional niche for wood-eaters, and these changes enable the development of xylophages.

## 1. Introduction

### 1.1. Nutritional Scarcity of Pure Wood

In terrestrial ecosystems, wood is an abundant but highly suboptimal food resource for heterotrophs because of its poor digestibility and nutrient deficiency. In dead wood, the *C:N* and *C:P* ratios (dry mass ratio/molar ratio) may be as high as 6500/7500 and 54,500/150,000, respectively [1,2,3,4,5,6], which indicates severe nutritional scarcity. The nutritional scarcity of dead wood poses severe stoichiometric mismatches for consumers (the proportion of C relative to other nutritional elements in the consumer’s body is much lower than in its food [7,8]). Based on literature data on the element content in wood [3,4,5] and insects [9,10,11,12], the estimated stoichiometric mismatch [7,8] may reach two orders of magnitude for living wood and three orders of magnitude for dead wood. This discrepancy can hamper or even prevent the growth and development of dead-wood eaters. Therefore it can be concluded that dead wood itself is too lacking in nutrients to produce the biomass of dead-wood eating beetles found in nature [1]. The numerous invertebrates that exploit this vast resource must depend on microbial support for not only cellulose, hemicellulose and lignin digestion, but also nutrient provisioning. The microbial digestion of cellulose and other sugars makes available only simpler compounds that still consist exclusively of C, H and O [13]. Thus, although symbiotic interactions of numerous xylophages with microorganisms may ease the digestibility of polysaccharides [14], the supply of nutritional elements cannot be increased in this manner. The only exception is nitrogen, which may be supplied by nitrogen-fixing organisms [15]. As a result, these symbionts cannot provide wood-eaters with their needed amounts of elements, which are found in extremely low total amounts in pure wood. Despite this, wood-boring beetle larvae somehow extract from wood stumps all of the elements necessary to grow and maintain their metabolism.

### 1.2. Nutritional Enrichment of Decomposing Wood Meets the Needs of Wood-Eaters

The larvae of wood-eating beetles (e.g., *Stictoleptura rubra*, *Arhopalus rusticus* and *Chalcophora mariana*) that inhabit pine stumps cannot gather sufficient elements for growth to maturity from wood alone. Therefore, it has been suggested that the nutrients needed by the beetles are supplied to pine stumps from the outside by fungi and that the beetles fulfill their nutritional needs by feeding on fungi as well as wood, which seems likely considering beetle consumption rate and development time [1]. The nutritional supplementation of dead wood can be thought of as a decrease in the ratio of C concentration relative to other elements concentrations as the wood decays. The concentrations of elements other than C may increase by up to two orders of magnitude during the first four years of decomposition (*i.e.*, the time corresponding to the development of xylophagous invertebrates) [1]. The most extreme stoichiometric mismatches reported to date (between xylophages and pure dead wood) can be substantially mitigated in this manner, allowing xylophages to develop. During the time period corresponding to the larval stage, xylophages need to gather total amounts of nutritional elements that are sufficient to build their bodies. This is possible only due to increases in the levels of elements other than C that are observed in decomposing wood. The development of beetles is primarily limited by the scarcity of N, P, Cu, K and Na in dead wood and the total amounts of these elements (except for Na) increase inside pine stumps during the wood decomposition process [1].

### 1.3. Searching for the Mechanisms Underlying Wood Enrichment During Decomposition

During the decomposition of coarse woody debris, the ratios of C to other elements decrease [2,3,4,5]. It is commonly assumed that the main factor responsible for these decreases in the ratios is the loss of C via respiration, supported by N fixation [2,6,16]. However, the increase in the concentrations of nutritional elements during wood decay cannot be explained solely by the release of carbon as CO_2_ [1]. Stark [17] demonstrated that nutritional elements are transported to the litter from the outside environment during decomposition, leading to the enrichment of decaying matter in scarce elements. A similar process may occur during wood decomposition. The enrichment of dead wood with biologically important elements other than C is significant and varies for different elements [2,3,4,18]. Such a variability could not be observed by considering the release of C from wood as the only cause of this enrichment. Possible candidates responsible for the nutritional enrichment of dead wood are fungi, which are capable of absorbing nutrients from both biotic and abiotic substrates and translocating them over long distances [16,19,20,21,22,23,24,25,26]; moreover, fungal tissues are rich in the elements that present the most marked increases in decaying wood [27,28,29,30]. It has been repeatedly proposed that fungi may alter the stoichiometry of dead wood. These proposals have been based both in theory [16,20] and in experimental practice [22]. The mechanism of the process is simple [16,17,19,20,21,22,23,24,25,26]: Fungal mycelia cover large areas of the forest floor and connect patches of the ecosystem that significantly differ in nutritional quality. While a portion of the mycelium penetrates dead wood, which is a good source of carbon, another part may absorb nutrients from decomposing organic matter or mineral soil outside of the dead wood. Ultimately, the acquired nutrients are translocated between the different areas through the fungal mycelium [16]. Nevertheless, the majority of studies considering the dynamics of nutritional elements during wood decay focused on the loss of nutrients during this process, on the leaching of nutrients from dead wood to the ecosystem, and on the late stages of dead wood decomposition when the transport of previously accumulated nutrients from dead wood to the outside may dominate [2,3,4,6,18,19,23]. Thus, the changes in the nutritional element concentrations that occur over a short time scale during the early stage of dead wood decomposition, corresponding to the development of xylophages, have not been taken into consideration [31]. In contrast, the xylophages that exploit pine stumps do so at the initial stages of wood decay, when substantial increases in the absolute amounts of nutritional elements apparently do occur [1]. These changes concern a whole array of nutritional elements that build molecules necessary for animals. The nutritional elements whose shortage can particularly limit xylophage development are N, P, K, Na and Cu [1], as determined based on the severe stoichiometric mismatches [7,8] between the food and growing tissues of wood-eaters. We hypothesize that during decomposition, fungi transport considerable amounts of specific nutritional elements (necessary for the development of wood-eaters) into dead wood, causing a rearrangement of the dead wood’s multi-elemental stoichiometry that allows xylophages to grow, develop, and reach maturity. This hypothesis can be corroborated by the existence of a dependency between the amount of fungal tissue in dead wood and the concentrations of nutritional elements other than C that are needed by developing wood-eaters. Conversely, a lack of such a dependency would falsify this hypothesis. Also, comparisons of changes in absolute amounts of elements during decay would be needed to determine whether the increase in fungal tissue or the liberation of C in the form of CO_2_ can exclusively explain the observed increases in the concentrations of elements during decay.

### 1.4. Aims of This Study

The aims of the present study were to determine whether the changes in the contents of 12 essential elements (C, N, P, S, K, Ca, Mg, Fe, Zn, Mn, Cu and Na) in decaying wood (pine stumps) can be attributed to the action of fungi, and to explain to what degree these changes meet the nutritional requirements of wood-eating beetles.

## 2. Materials and Methods

### 2.1. Dead-Wood Nutritional Enrichment

We collected pine stumps (*Pinus sylvestris*) in various stages of decay (approx. five months to five years after tree cutting) from approximately 80-year-old pine stands in the Niepołomice Forest (Puszcza Niepołomicka, southern Poland, 50°05′N, 20°21′E, elevation 184–212 m above sea level). Seventy-seven stumps were sampled randomly across a forested area of approximately 110 km^2^, with the date of tree cutting known for most stumps. The stumps were sampled whole at ground level. The stumps were hand split to collect samples of dead wood and were subsequently ground and homogenized. The wood samples collected for chemical analysis contained both heartwood and sapwood but not bark. The stumps were inhabited by larvae of three xylophagous beetles species that are considered to be “dead-wood eaters”: *Stictoleptura rubra* Linnaeus 1758 (=*Corymbia rubra* Nakano and Obayashi 1957; =*Aredolpona rubra* Viliers 1974), *Arhopalus rusticus* Linnaeus 1758 (=*Criocephalus rusticus* Haldeman 1847; Coleoptera, Cerambycidae), and *Chalcophora mariana* Linnaeus 1758 (=*Buprestis mariana* Linnaeus 1758; Coleoptera, Buprestidae).

The wood samples were freeze-dried to allow dry mass measurements without the destruction of organic compounds. Subsamples were obtained from each sample for specific analyses. The element concentrations were determined by atomic absorption spectrometry (Perkin-Elmer AAnalyst 200 and Perkin-Elmer AAnalyst 800), colorimetrically (MLE FIA flow injection analyzer), and by using an automatic CHNS analyzer (Vario EL III). The measurement procedures were described in detail by Filipiak and Weiner [1]. For the elemental analysis, the averages of two subsamples from each stump were calculated. The ergosterol content was measured in samples extracted from wood (one subsample per stump) following standard procedures [32] using a Clarus 600 GC/MS chromatograph. Ergosterol is a specific biomarker for fungi (although small amounts of ergosterol can also be found in algae and protozoa), and its use as a proxy for the amount of fungal mycelia is the widely accepted and most dependable method of quantifying fungal biomass [33,34,35].

A multivariate redundancy analysis (*RDA*) was performed on log-transformed and standardized data (treating the element content as the dependent variable and the ergosterol content as the independent variable).

**Figure 1:**
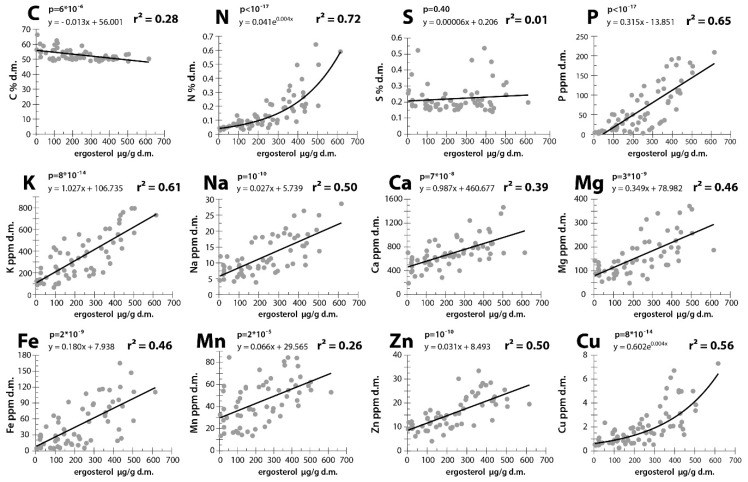
Relationships between ergosterol content and the levels of nutritional elements in dead wood.

To evaluate the relationships between the ergosterol and element contents (linear or non-linear regression), we calculated *r^2^*. To select the model (linear *vs.* non-linear regression) that better explained the observed relationship for each element, we additionally calculated the Akaike information criterion (*AIC*). Based on this, we determined the appropriate model (linear *vs.* non-linear regression) for each element, as shown in Figure 1. Bonferroni correction was performed on the *p*-values of the regressions.

To determine whether the increase in fungal tissue or C loss (respiration) exclusively is sufficient to explain the observed increases in the concentrations of elements and ergosterol during decay, it is necessary to compare the changes in the absolute amounts of the different elements and ergosterol during the course of decay. This analysis can be performed using information on the decrease in dead wood density obtained as the decay process proceeds during the five years of decomposition studied. The easiest way to compare the changes in the absolute amounts of elements is through the simple calculation of the “coefficient of difference” *β* (*cf*. “coefficient of enrichment” *β*, [1]), which is defined as

(1) $$x_{F}=x_{I}\times \beta$$

where *x_F_* is the final absolute amount of element *x* or ergosterol in a stump after a given time of decomposition, and *x_I_* is the initial absolute amount of element *x* or ergosterol in a stump at the beginning of the study. From equation (1), it follows that

(2) $$\beta =\frac{x_{F}}{x_{I}}=\frac{X_{F}\times M_{F}}{X_{I}\times M_{I}}=\left(\frac{X_{F}}{X_{I}}\right)\times \left(\frac{M_{F}}{M_{I}}\right)$$

where *x*_I_ and *x*_F_ are the initial and final absolute amounts of element *x* or ergosterol, respectively, *X*_I_ and *X*_F_ represent the initial and final concentrations (as dry mass % or ppm) of element *x* or ergosterol, respectively, and *M*_I_ and *M*_F_ are the initial and final masses of the decaying stump, respectively. In the considered case, initial refers to the lowest amount of fungal tissue, which means that the stump is in its initial stage of decay (*i.e.*, within the first year after tree cutting), whereas final refers to the highest amount of fungal tissue, which means that the stumps were highly decayed and analyzed five years after tree cutting. The *X*_F_/*X*_I_ ratio may be calculated from the data obtained in this study, and the *M*_F_*/M*_I_ ratio can be estimated from literature data as described below.

Equation (2) shows the change in the absolute amount of element *x* or ergosterol that occurred during a given time (five years in this study) of decay and fungal infection. An increase in the absolute amount of element *x* in a decaying stump results in values of *β > 1*. For the purpose of this study, to avoid overestimation resulting from generalizations, we conservatively assumed that *β >> 1* instead of *β > 1*. Therefore, in this study, *β > 2* indicates the transport of a specific nutritional element from the outside environment of the stump during the decay process.

The aim of the study was to determine which nutritional elements (if any) may be transported by fungi to decaying stumps, and to what degree this occurs (if at all). Therefore, we selected for our calculations those stumps with the most extreme (five of lowest and five of highest) ergosterol concentrations. Five stumps were selected to represent the initial stage of fungal infection (*i.e.*, the initial stage of decomposition). These stumps had aged less than one year after tree cutting and had ergosterol concentrations less than 25 µg/g d.m. To represent the final stage of decomposition, which is characterized by high concentrations of fungi, we used five stumps with ergosterol concentrations greater than 440 µg/g d.m. (see supplement: Table S1). These stumps had aged five years after tree cutting. Using the selected stumps, we calculated the average concentrations of the different elements and ergosterol at the initial and final stages. The unknown proportion *M*_F_*/M*_I_ can be approximated from data in the literature concerning the rates of decay of coarse woody debris (e.g., [36,37,38,39]) using the exponential model. *M*_F_/*M*_I_ = *e*^−*k*t^. According to the literature, the experimentally evaluated constant *k* ranged from 0.0200 to 0.1101, depending on the tree taxon, environmental conditions, and methods used [36,37,38,39]. Therefore, we used the two most extreme values of *k* to calculate possible extreme values of *β*. The model solved for five years of decay using these values yielded a proportion of remaining mass between 0.58 and 0.90.

Using this procedure, we could estimate the direction of changes (no change/decrease/increase) in the absolute amounts of various elements and ergosterol in decomposing stumps based on the measured relative concentrations of the studied elements.

### 2.2. Nutritional Limitation of Xylophages

To express how the xylophagous beetles (“wood-eaters”) inhabiting pine stumps depend on the nutritional enrichment of dead wood, we used previously published data on the elemental composition of *Stictoleptura rubra*, *Arhopalus rusticus* and *Chalcophora mariana* imagines of both sexes [1] and on the elemental composition of the pine stumps sampled here. We calculated the stoichiometric mismatches experienced by beetles feeding on the 10 stumps that were used to calculate *β* (for every element concentration we used the mean concentration calculated for the five stumps with the lowest and the five stumps with the highest ergosterol concentrations). We expressed the degree of the stoichiometric mismatches for each element *x* as the ratio of the stoichiometric ratios in the food relative to the consumer’s body (Trophic Stoichiometric Ratio = *TSR*; *cf*. [1]):

$$TSR_{X}=\frac{{\left(C:X\right)}_{food}}{{\left(C:X\right)}_{consumer}}$$

where C refers to the carbon content and *X* refers to the content of element *x*.

This index does not depend on the units used for stoichiometric ratios *C:x* (molar or mass units). Values of *TSR_X_* >> 1 indicate stoichiometric mismatches, with severe mismatches indicated by *TSR* values substantially different from unity. For the purpose of the study, to avoid overestimation [40,41,42], we assume that a *TSR_x_* ≥ 10 indicates a stoichiometric mismatch posing constraints on the development of the studied beetles (*cf*. Threshold Elemental Ratio *TER* [7,8,40]).

We express the degree of stoichiometric mismatch mitigation with fungal action as follows:

$$\alpha =\frac{TSR_{L}}{TSR_{H}}$$

where α refers to the degree of stoichiometric mismatch mitigation with fungal action; *TSR_L_* refers to the mean *TSR* ratio calculated from the *TSR* ratios for the three studied beetle species feeding on the five stumps with the lowest ergosterol concentrations; *TSR_H_* refers to the mean *TSR* ratio calculated from the *TSR* ratios for the three studied beetle species feeding on the five stumps with the highest ergosterol concentrations.

## 3. Results

All species and sexes of the studied beetles would experience extremely high stoichiometric mismatches while feeding on stumps with the lowest concentrations of ergosterol (*i.e.*, the lowest level of fungal infection): the *TSR_I_* ratios were greater than 1000 for P, greater than 100 for N and Na, and greater than 10 for K, Mg and Cu (Table 1). These mismatches were considerably mitigated when it was assumed that the beetles were feeding on the stumps with the highest ergosterol concentrations (*i.e.*, the highest level of fungal infection): *α* reached nearly 50 for P, 13 for N, and nearly 10 for Fe, Cu and K. For the other elements, the mitigating effects were low (α ≤ 3.4, Table 1).

The concentrations of all of the elements studied, with the exception of C and S, increased with increasing ergosterol concentrations (Figure 1). Exponential regressions provided a better fit than linear regressions for N and Cu, whereas for the remaining elements, these two models yielded similar results. The carbon concentration decreased with an increase in the ergosterol content (*r^2^* = 0.28), whereas the S concentration was not related to the ergosterol content (Figure 1). The positive relationship between the ergosterol and element contents in decomposing wood was strongest for N (*r^2^* = 0.72), followed by P (*r^2^* = 0.65), K (*r^2^* = 0.61) and Cu (*r^2^* = 0.56, Figure 1). This relationship was also moderately strong for Na (*r^2^* = 0.50), Zn (*r^2^* = 0.50), Fe (*r^2^* = 0.46) and Mg (*r^2^* = 0.46). The weakest positive interdependence was observed for Ca (*r^2^* = 0.39) and Mn (*r^2^* = 0.26). After a Bonferroni correction, the p-values of the regressions were significant for all elements except S.

**Figure 2:**
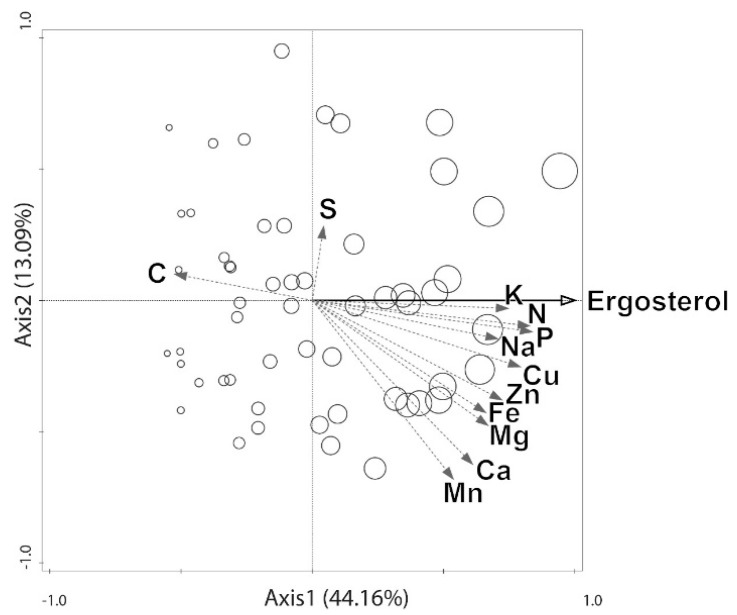
Multivariate analysis of the stoichiometric relationships between ergosterol content and the 12 studied elements. The *RDA* plot and the first two axes are shown (the circle size indicates ergosterol content).

A redundancy analysis (*RDA*) allowed the simultaneous comparison of the compositions of multiple elements in the wood and their relationship with the ergosterol content (Figure 2). The first and second axes explained 44.2% and 13.1% of the total variance, respectively (together, 57.3%). Thus, the ergosterol concentration adequately explains the pattern shown in Figure 2. The first axis was significant (*p* = 0.002), indicating a relationship between the ergosterol content and the element concentrations. The strongest positive correlations with ergosterol were obtained for the concentrations of P (1st axis loading: 0.83), N (0.82), Cu (0.78), K (0.78) and Zn (0.78). The 1st axis loadings for Ca and Mn were less than 0.70 (0.60 and 0.50, respectively), and the S concentration displayed no correlation with the ergosterol content (1st axis loading: 0.04). The C concentration was negatively, but weakly, correlated with the ergosterol content (1st axis loading: −0.52).

Considering the nutritional needs of wood-eaters, we also evaluated the relationships between the ergosterol content and the *C:X* (*X* = concentration of an element other than C) and *N:X* (*X* = concentration of an element other than C and N) ratios in a manner similar to that used for the element concentrations (detailed data in supplement). The *C:X* ratios (except *C:S*) were significantly and negatively correlated with the ergosterol content and were best fit with exponential regressions (higher *r^2^* and lower *AIC* values than the linear regressions). The relationships were strong for *C:N* (*r^2^* = 0.73), *C:P* (*r^2^* = 0.61), *C:Cu* (*r^2^* = 0.60), *C:K* (*r^2^* = 0.56), *C:Zn* (*r^2^* = 0.55), and *C:Na* (*r^2^* = 0.53), weaker for *C:Mg* (*r^2^* = 0.47), *C:Fe* (*r^2^* = 0.46) and *C:Ca* (*r^2^* = 0.41), and weakest for *C:Mn* (*r^2^* = 0.34).

No correlations were found between the ergosterol content and the *N:Cu* and *N:Fe* ratios, whereas positive correlations were observed for other *N:X* ratios. For *N:S* and *N:P*, exponential regressions yielded a better fit than linear regressions. The relationship was strongest for *N:S* (*r^2^* = 0.59), moderately strong for *N:Ca* (*r^2^* = 0.46) and *N:Zn* (*r^2^* = 0.41), weaker for *N:Mn* (*r^2^* = 0.36), *N:Mg* (*r^2^* = 0.33), and *N:Na* (*r^2^* = 0.32), and weakest for *N:P* (*r^2^* = 0.18) and *N:K* (*r^2^* = 0.14).

The estimated contribution of transport from outside of the stump to the observed increase in non-C element and ergosterol concentrations (Table 2) shows that independently of the assumed value of *M*_F_/*M*_I_, the amounts of S, Ca, Zn, and Mn do not increase during fungal infection (coefficients of difference do not deviate substantially from 1.0, ranging from 0.6 to 1.7 for *M*_F_/*M*_I_ = 0.58 and from 0.9 to 1.9 for *M*_F_/*M*_I_ = 0.90), whereas the levels of ergosterol, P, N, Cu, Fe and K increase several fold (Table 2); an increase in the absolute amounts of Na and Mg is indicated only when assuming *M*_F_/*M*_I_ = 0.9. Thus, changes in the relative concentrations of ergosterol, P, N, Cu, Fe and K result from the net import of these nutrients from outside the system and not solely from the loss of carbon. Carbon loss during decomposition is indicated by a coefficient of difference *β* with values in the range of 0.5 to 0.8 (Table 2).

## 4. Discussion

### 4.1. Elements That May be Transported by Fungi to Dead Wood From the Outside Environment

The levels of most of the elements investigated in this study were correlated with the ergosterol content of the dead wood (Figure 1 and Figure 2). The elements that were most strongly associated with ergosterol content were N, P, K, Cu and Na (Figure 1 and Figure 2). The observed correlations resulted from increases in the total amounts of ergosterol and elements, and not solely from C loss (respiration), as indicated by the “coefficient of difference” *β* (Table 2). A possible alternative explanation for the correlations between ergosterol content and the element concentrations, namely that fungi grow faster on decaying wood that is richer in nutritional elements, seems unlikely, considering there is no known process other than fungal action that could explain the translocation of considerable amounts of P, K, Ca, Mg, Fe, Zn, Mn, Cu and Na into dead wood from external nutritional pools. Nutrients and ions could be imported by fungi into the dead wood from outside by translocation through fungal mycelia that penetrate and decompose nutritionally rich organic matter as well as inorganic sources of ions (minerals and rocks). In this way, fungi can connect regions that are distant from one another and translocate nutrients and ions between them [16,17,19,20,21,22,23,24,25,26]. The amounts of elements being imported into decaying wood by fungi can exceed the amounts currently present in living fungal tissue and may be stored as fungal metabolites and decaying dead mycelium, whereas the ergosterol associated with dead fungi is subject to decomposition (35% decrease in concentration over two months [33]). By contrast, only N, and no other elements, can be imported through microbial nitrogen fixation [15,21]. Therefore, the correlations between fungal mass and element concentrations can result in non-linear (exponential) regressions (Figure 1). Indeed, the ability of fungi to translocate major nutrients has been documented in previous studies [16,19,21,22].

### 4.2. Nutritional Enrichment of Dead Wood by Fungi Allows for the Development of Wood-Eaters

Because the translocation of elements leads to changes in the stoichiometry of dead wood and lowers the stoichiometric mismatch between dead wood and its decomposers, fungi create a nutritional niche for saprotrophic (or xylophagous) arthropods from a habitat that was nutritionally too scarce to be inhabited. We previously determined that N, P, Cu, K and Na are the most strongly limiting elements for the development of xylophagous beetles that are involved in the dead wood decomposition process (*S. rubra*, *A. rusticus*, *C. mariana* [1]). These elements are largely delivered by fungi (Table 1 and Table 2 and Figure 1 and Figure 2) which considerably mitigates the limitation (Table 1). In addition, the ratios of these elements remain largely constant (constant *N:Cu* and *r^2^* < 0.2 for regressions relating the *N:P* and *N:K* ratios to the ergosterol content, but the *N:Na* ratio is definitely not constant). The almost constant *N:P* and *N:K* ratios and constant *N:Cu* ratio suggest that fungal action may cause a stable supply of the most important nutritional elements during the progress of decomposition while continuously reducing the stoichiometric mismatch for xylophages. This finding indicates that elements crucial for building the molecules needed for the development of wood-eaters are being delivered to wood-eaters at constant proportions throughout their development period. In addition, the availability of these elements increases during the development of xylophages (*i.e.*, the total amounts of these elements increase). As a result, wood-eaters experience a stable supply of the matter they most need for development and are able to maintain a stable assimilation of these nutrients. Other elements delivered by fungi include Mg, Zn and Fe, which are also scarce in dead wood, and shortages of these elements may slow the developmental time of xylophages (Table 1 and Table 2 and Figure 1 and Figure 2). We calculated to what degree fungal action may alter the *C:X* (*C* = concentration of carbon, *X* = concentration of every non-carbon element *x* studied) ratios and thus reduce the stoichiometric mismatches experienced by wood-eaters (*Stictoleptura rubra*, *Arhopalus rusticus*, and *Chalcopora mariana*, Table 1). The ratios for P, N, Fe, Cu, K, and the remaining elements were reduced by a factor of 48, 13, 9, 8, 7, and two or three-fold, respectively (Table 1). This alteration in stoichiometry is necessary for the infestation of dead wood by wood-eaters because it is the only possible mechanism that would allow these organisms to cope with the extremely low stoichiometric mismatches observed in dead wood [1]. However, even if reduced by fungal action, stoichiometric mismatches still occur for certain elements (N, P, Na and K, Table 1). This scarcity constrains beetle development to some degree, which may be overcome through prolonged larval development time, as observed in nature [1]. This is also aided by the relatively low mortality rate for xylophages living inside dead wood [43,44]. Furthermore, selective feeding on patches of dead wood that are heavily infested by fungi could additionally mitigate this limitation.

### 4.3. Nutrient Cycling Pathways and Dead Wood Fungi

Our findings partially support the theoretical predictions made by Swift *et al*. [20] and Boddy and Watkinson [16], *i.e*., the decrease in the ratios of C relative to other elements does occur during dead wood decomposition, but the strength of the decrease varies according to the element and is shaped by the increase in the total mass of non-carbon elements. We emphasize the direction of the fungal transport of nutrients that occurs in the studied system during the first five years of decomposition. Scarce elements that limit the development of wood-eaters are transported to the dead wood from outside and not taken from the wood as previously suggested [2,6,16,23,31]. The action of xylophages ease and speed up the decomposition process [45,46]. As a result, fungi trigger the decomposition process of dead wood via changes in the dead wood stoichiometry during decay.

## 5. Conclusions

It can be concluded that a direct comparison of the amounts of essential nutrients and ergosterol in wood provides evidence that the changes in the stoichiometry of decaying dead wood are driven by the action of fungi that infest dead wood to take advantage of the ample supply of an energy-rich substrate. However, to exploit this resource, these fungi must import an array of essential nutrients from outside of the dead wood. This nutritional enrichment of dead wood creates a nutritional niche for xylophages that allows them to grow, develop, and reach maturity. Therefore, xylophagous beetles (considered as “wood-eaters”) are unable to gather the necessary amounts of nutritional elements from pure dead wood to grow and mature, but instead must utilize fungal tissues.

## Figures and Tables

**Table 1:** Nutritional limitation of xylophagous beetles expressed as Trophic Stoichiometric Ratios (*TSRs*) ((*C:x)_food_/(C:x)_consumer_*).

	Stoichiometric Mismatch (*TSR*)	N	P	K	Na	Ca	Mg	Fe	Zn	Mn	Cu
**Females**	*S. rubra TSR_L_*	258.6	1772.9	70.6	133.5	2.6	15.9	11.0	11.6	0.7	78.7
	*S. rubra TSR_H_*	19.5	36.9	9.7	39.8	1.0	5.7	1.2	5.1	0.4	9.3
	*A. rusticus TSR_L_*	133.3	1252.1	54.8	104.7	1.6	12.9	6.2	12.6	0.5	68.5
	*A. rusticus TSR_H_*	10.0	26.1	7.5	31.2	0.7	4.6	0.7	5.5	0.2	8.1
	*C. mariana TSR_L_*	207.5	1790.2	89.0	85.2	2.1	25.4	2.8	8.0	0.8	12.2
	*C. mariana TSR_H_*	15.6	37.3	12.2	25.4	0.9	9.1	0.3	3.5	0.4	1.5
**Males**	*S. rubra TSR_L_*	206.3	1052.2	54.6	101.5	2.1	13.8	3.9	10.7	0.8	30.7
	*S. rubra TSR_H_*	15.5	21.9	7.5	30.3	0.8	4.9	0.4	4.7	0.4	3.6
	*A. rusticus TSR_L_*	177.6	1458.0	72.9	90.7	1.7	20.3	3.3	7.5	0.9	11.7
	*A. rusticus TSR_H_*	13.4	30.3	10.0	27.1	0.7	7.3	0.4	3.3	0.4	1.4
	*C. mariana TSR_L_*	244.4	1669.5	70.3	176.2	1.9	18.3	4.1	8.7	0.7	15.2
	*C. mariana TSR_H_*	18.4	34.7	9.6	52.6	0.8	6.6	0.5	3.8	0.3	1.8
	α	13.3	48.1	7.3	3.4	2.5	2.8	9.1	2.3	2.1	8.4

*TSR_L_*––ratio calculated for the stumps with the lowest ergosterol concentrations; *TSR_H_*––ratio calculated for the stumps with the highest ergosterol concentrations; α––degree of stoichiometric mismatch mitigation due to fungal action (mean *TSR_L_*/mean *TSR_H_*). See text for details. The beetles are nutritionally limited while feeding on stumps with the lowest ergosterol content. Fungal action considerably mitigates this limitation and greatly reduces the experienced mismatches for the most limiting elements (P, N, Cu and K).

**Table 2:** Coefficient of enrichment (*β*) of elements and ergosterol in wood inhabited by fungi.

**Value of *M*_F_*/M*_I_**	**Values of *β* for the Measured Elements and Ergosterol**												
	C	**N**	S	**P**	**K**	Na	Ca	Mg	**Fe**	Zn	Mn	**Cu**	**erg**
0.90	0.8	**10.3**	0.9	**37.3**	**5.7**	**2.6**	1.9	**2.2**	**7.0**	1.8	1.6	**6.5**	**28.8**
0.58	0.5	**6.6**	0.6	**24.0**	**3.7**	1.7	1.2	1.4	**4.5**	1.1	1.0	**4.2**	**18.6**

Bold letters indicate coefficients of enrichment exceeding 2, which are indicative of elements and ergosterol imported to the decaying wood from outside. *M*_F_*/M*_I_ refers to the ratio of the final to the initial mass of the stump = the percentage of mass of the stump that was not decomposed during the considered time (5 years); erg refers to ergosterol. The extreme (lowest and highest) values of *M*_F_*/M*_I_ were used to calculate the possible extreme values of *β*. Both values indicate that P, Fe, N, Cu and K are being transported by fungi to the decaying stumps.

## Supplementary Materials

The following are available online at www.mdpi.com/2075-4450/7/2/13/s1, Figure S1: Relations between ergosterol contents and: (**A**) ratios *C: other elements*; (**B**) ratios *N: other elements*, excluding *N: C*. Measured concentrations and calculated ratios concern wood samples from pine stumps at various stages of decomposition (approx. five months to five years after tree cutting), Table S1: All raw data collected. For *RDA* analysis, only samples containing the whole dataset (12 elements + ergosterol contents) were used.

## Abbreviations

The following abbreviations are used in this manuscript:

*RDA*: Multivariate redundancy analysis
*AIC*: Akaike information criterion
