# Supplementary material for: Fungal Transformation of Tree Stumps into a Suitable Resource for Xylophagous Beetles via Changes in Elemental Ratios
Source: Insects. 2016 Apr 9;7(2):13. doi: 10.3390/insects7020013 (PMC4931425; doi:10.3390/insects7020013)
Supplement: Supplementary file 1 [file insects-07-00013-s001.zip › insects-120306-Figure S1.pdf]

Supplementary Material

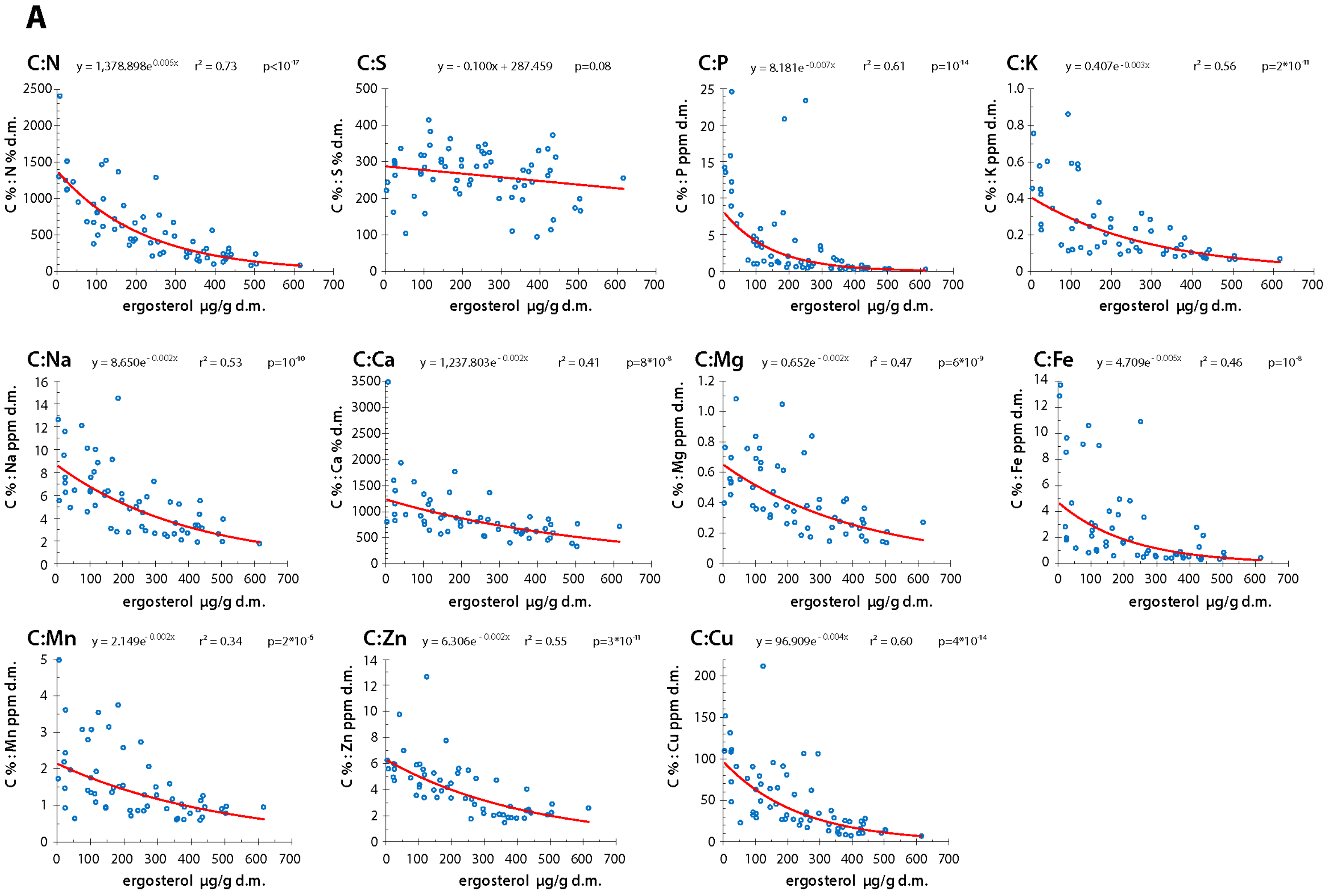

Figure S1. *Cont.*

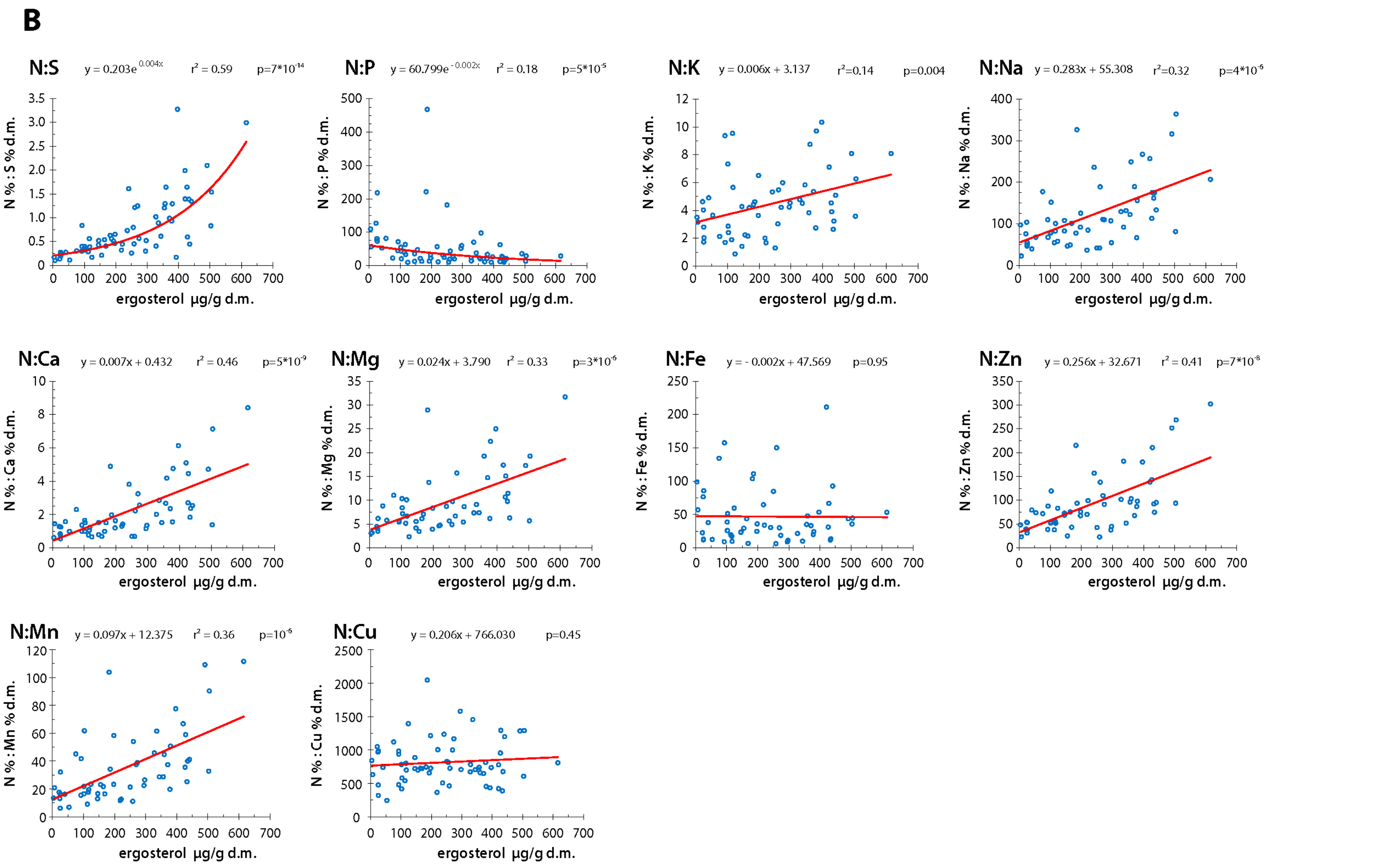

Figure S1. Relations between ergosterol contents and: (**A**) ratios *C: other elements*; (**B**) ratios *N: other elements*, excluding *N: C*. Measured concentrations and calculated ratios concern wood samples from pine stumps at various stages of decomposition (approx. five months to five years after tree cutting).
